# Reaction Coordinates for Conformational Transitions Using Linear Discriminant Analysis on Positions

**DOI:** 10.1021/acs.jctc.3c00051

**Published:** 2023-05-02

**Authors:** Subarna Sasmal, Martin McCullagh, Glen M. Hocky

**Affiliations:** † Department of Chemistry and Simons Center for Computational Physical Chemistry, New York University, New York, New York 10003, United States; ‡ Department of Chemistry, Oklahoma State University, Stillwater, Oklahoma 74078, United States

## Abstract

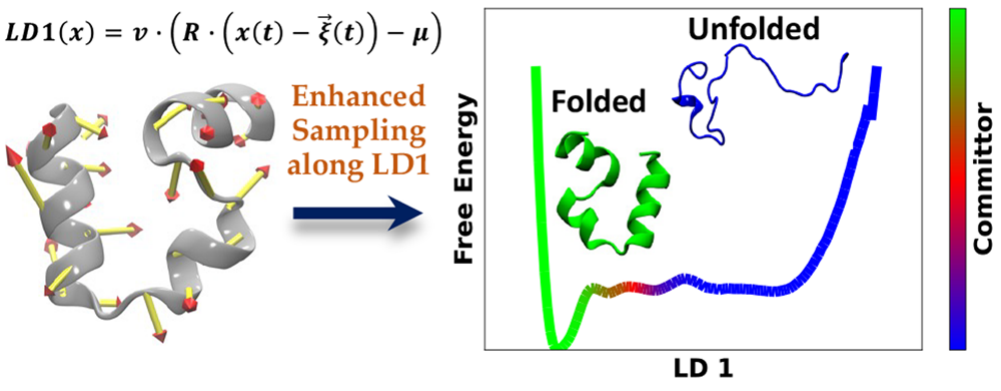

In this work, we demonstrate that Linear Discriminant Analysis (LDA) applied to atomic positions in two different states of a biomolecule produces a good reaction coordinate between those two states. Atomic coordinates of a macromolecule are a direct representation of a macromolecular configuration, and yet, they are not used in enhanced sampling studies due to a lack of rotational and translational invariance. We resolve this issue using the technique of our prior work, whereby a molecular configuration is considered a member of an equivalence class in size-and-shape space, which is the set of all configurations that can be translated and rotated to a single point within a reference multivariate Gaussian distribution characterizing a single molecular state. The reaction coordinates produced by LDA applied to positions are shown to be good reaction coordinates both in terms of characterizing the transition between two states of a system within a long molecular dynamics (MD) simulation and also ones that allow us to readily produce free energy estimates along that reaction coordinate using enhanced sampling MD techniques.

## 1 Introduction

Many enhanced sampling techniques work by biasing a system to explore along a low dimensional set of collective variables (CVs).^1^ These methods allow us, in principle, to use the known applied bias to reconstruct the free energy landscape in that low dimensional space. In practice, the choice of the CVs is crucial, with an ideal set of CVs allowing the system to explore all relevant states within available simulation time.^1^ Recently, extensive effort has been invested in using a variety of machine learning approaches, from very simple to very sophisticated, to determine optimal coordinates for sampling from molecular dynamics (MD) simulation data (refs (2−21) provide a representative but not exhaustive sample).

One commonly encountered challenge is to compute the free energy path of a transition between two states along a linear dimension that chemists term a reaction coordinate (RC). For a macromolecule such as a protein, the two states could be configurations for which we have known structures (e.g., the PDB structure of a protein solved with and without a bound ligand) or processes for which one state is known and the other state can be at least qualitatively defined (e.g., folding/unfolding or binding/unbinding). If a long MD trajectory containing multiple transitions between these states is available, then reaction coordinates could be trained based on the idea that we want to enhance sampling along the slowest modes in the system.^4,10,13,14,22,23^ However, having this data is rare, in which case one can try iterating sampling and learning reaction coordinates with the goal of maximizing the number of transitions between the two states in a fixed amount of simulation time.^4,5,11,13,15,24^

An alternative approach which has shown some success is to train reaction coordinates based on short simulations within the two states and use a method that produces a coordinate representing the difference between the two sets of data. Linear dimensionality reduction techniques such as Principal Component Analysis (PCA) and Linear Discriminant Analysis (LDA) are the simplest approaches for combining a large set of variables that describe a system of interest to produce a small set of CVs that characterize the available data. While PCA, which produces coordinates that capture the most variance in the data, has been used to promote exploration in enhanced sampling simulations, LDA seems to hold more promise as an RC since it is a supervised approach designed to maximally separate different labeled classes of data (i.e., reactants and products). We describe LDA in full detail in the next section. In one study, Mendels et al.^6^ produced a modified approach to LDA termed harmonic LDA (HLDA, because the covariance matrices in the two different states of interest are combined by a harmonic average rather than a simple sum) and, in that work and subsequent ones,^7,9^ combined it with Metadynamics (MetaD) to effectively enhance sampling between two states for several different systems. Later, a neural network was used to combine features before training LDA vectors to produce the reaction coordinate.^16^

In the prior examples of reaction coordinate design for free energy sampling of biomolecules that we are aware of, the input features to the method were internal coordinates, or a function of internal coordinates, for the molecule(s) of interest—for example, distances, angles, and dihedrals. Often, these could be CVs based not on atomic positions directly but on coarse-grained (CG) representations of the biomolecule, such as the distance between the centers of masses (COMs) of two different domains or the distance between the COM of a ligand and certain atoms in its binding pocket. This is not surprising, because these often correspond to our physical intuition about the biomolecular reaction coordinate. Moreover, internal coordinates are invariant to translation and rotation of the molecule, and thus bias forces applied to these coordinates do not depend on the position or orientation of the molecule.

Recently, we presented atomic coordinates as an alternative set of features to use in the context of clustering biomolecular data.^25^ Atomic coordinates of a subset of atoms, or of beads corresponding to a CG representation of a molecule, offer an alternative to internal coordinates with the advantage that there is little choice in selecting the features to use. Using a protein as an example, we need only make the standard choice between C_α_ atoms, backbone, all heavy atoms, and so on. Moreover, only 3*N* – 6 atomic coordinates essentially describe the state of a biomolecular system with *N* important atoms (but ignoring contributions of solvent, salt, etc.), whereas use of internal coordinates often results in an overdetermined set of features, such as all *O*(*N*^2^) pairs of distances. In ref (25), we developed a procedure for clustering molecular configurations into a Gaussian mixture model (GMM) using atomic positions that overcomes challenges of orientational dependence that prevented their use earlier, as described below. Because a Gaussian mixture model in positions is a natural way to coarse-grain a free energy landscape,^25−28^ with locally harmonic bins around metastable states, the resulting clustering is a physically appealing definition of the “states” a molecule can adopt.

However, our Gaussian mixture model still relies on a very high (3*N* – 6) dimensional representation of our molecule. Given that the output of our clustering algorithm is a set of states each defined by a multivariate Gaussian distribution, LDA is a natural approach to produce a low dimensional representation of our data with large separation between states. In this work, we first apply LDA to the folded and unfolded states determined from shapeGMM clustering of a long unbiased MD trajectory of a fast-folding protein and demonstrate that it produces a physically reasonable ordering of states from folded to unfolded. We then show that this coordinate is a “good” reaction coordinate because the position of the barrier separating folded and unfolded is very close to the location where the system is equally likely to proceed to folded or unfolded (in terms of a committor function to be defined below). We implement this position LDA coordinate in the PLUMED sampling library and demonstrate that biased sampling along this coordinate can accelerate transitions between the folded and unfolded states and produce a qualitatively similar free energy surface as compared to the unbiased trajectory in 3% of the simulation time, without any additional tuning of the CV. Finally, we train a position LDA coordinate on an achiral helical system where data is only available in the left- and right-handed states and show that this coordinate also allows us to readily sample between the two states, despite there being no information about the transition provided during training.

## 2 Theory and Methods

### 2.1 Molecules in Size-and-Shape Space

Consistent with our previous work on structural alignment and clustering,^25^ we consider structures from an MD simulation to be associated with Gaussian distributions in atomic positions. Structures are represented by *N* particles (a subset of atoms) using a vector ***x*** of dimensions *N* × 3 which is a member of an equivalence class
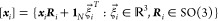
1where ξ⃗_*i*_ is a translation in , ***R***_*i*_ is a rotation , and **1**_*N*_ is the *N* × 1 vector of ones. [***x***_*i*_] is a point in size-and-shape space^29^ which has dimension 3*N* – 6 and is defined as  where  is the group of all rigid-body transformations for each frame with elements ***g*** = (ξ⃗, ***R***).

Within the shapeGMM framework, the probability density of particle positions is assumed to be a Gaussian mixture
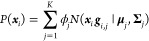
2where *N*(***x***_*i*_ ***g***_*i*,*j*_|**μ**_*j*_, **Σ**_*j*_) is the *j*th normalized, multivariate Gaussian with mean **μ**_*j*_, covariance matrix **Σ**_*j*_, and weight ϕ_*j*_ (the weights are normalized such that ∑_*j*=1_^*K*^ ϕ_*j*_ = 1). ***g***_*i*,*j*_ is the element of *G* that minimizes the Mahalanobis distance between ***x***_*i*_ and **μ**_*j*_. Iterative determination of ***g***_*i*,*j*_ and **μ**_*j*_ is performed in a Maximum Likelihood procedure.^25^

In the current work, we will consider LDA coordinates learned using data from only two states. Additionally, we will only consider “weighted” alignment of particle positions, which equates to using a Kronecker product covariance (where Σ_*j*_ = Σ_*N*_ ⊗ *I*_3_, for Σ_*N*_ the *N* × *N* covariance of particle positions) in defining the Mahalanobis distance between frame and average structure as described in detail in ref (25).

### 2.2 Dimensionality Reduction Using Linear Discriminant Analysis on Particle Positions

We propose to use LDA directly on aligned particle positions as a reaction coordinate. LDA for two states produces the linear model with the maximal interaverage variance while minimizing intracluster variance.^30^ For *K* different clusters, this is achieved by first computing the within-cluster scatter matrix
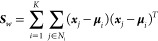
3and the between-cluster scatter matrix
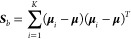
4where **μ**_*i*_ is the average structure of cluster *i*, and **μ** is the global average. The simultaneous minimization of within-cluster scatter and maximization of between cluster scatter can be achieved by finding the transformation *G* that maximizes the quantity
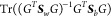
5This maximization can be achieved through an eigenvalue/eigenvector decomposition, but such a procedure is only applicable when ***S***_*w*_ is nonsingular. The LDA method was reformulated in terms of the generalized singular value decomposition (SVD)^31^ extending the applicability of the method to singular ***S***_*w*_ matrices such as those encountered when using particle positions.

In addition to employing the SVD solution to the LDA approach, care must be taken in how particle positions are aligned when performing LDA. This is evident when one considers the scatter matrices in eq 3 and eq 4. The values and null spaces of these scatter matrices will depend on the specific alignment procedure chosen. There are three obvious choices for structural alignment prior to LDA: (1) alignment of each frame to its respective cluster mean/covariance, (2) alignment to one cluster or another, and (3) alignment to a global average. The first choice will lead to scatter matrices with different null spaces for each cluster making their addition in eq 3 unsatisfactory. Alignment to a cluster mean will yield consistent null spaces for each cluster but requires distinct alignment reference and global average structures. Additionally, aligning to a cluster mean yields to an undesirable ambiguity (and asymmetry) in the choice of cluster. Alignment to a single global average overcomes all of these issues and, as we show in the Supporting Information (Sec. S6), yields a sampling coordinate that is at least as good as alignment to a cluster mean for the systems tested here.

The result of an LDA procedure on two labeled states will be a vector, ***v***, of coefficients that best separate the two states. These vectors are similar in nature to the eigenvectors from PCA, a procedure more familiar to the biosimulation field.

### 2.3 Biasing a Linear Combination of Positions

The value of the LDA coordinate after this procedure is a dot product of the vector ***v*** with the atomic coordinates ***x*** – **μ**. When computing this value on the fly within an MD simulation, we need to consider the value of [***x***(***t***)], the equivalence class of the position at time *t*, translated and rotated to a reference {**μ**, **Σ**}.

Therefore, to compute the value of the LDA coordinate *l*, we first translate ***x***(***t***) by , the difference in the geometric mean of the current frame and that of the reference configuration. Then, we compute ***R***(*t*), the rotation matrix which minimizes the Mahalanobis difference between ***x***(***t***) – ξ⃗ and **μ**, for a given **Σ**, as described in ref (25). Finally, we compute
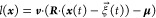
6By definition, *l*(**μ**) = 0.

To apply bias forces to this coordinate, we must be able to compute ∇*l*(***x***(t)). Because of the inclusion of the optimal rotation process by SVD, it is nontrivial to compute this analytically, and we instead compute derivatives numerically.

### 2.4 Enhanced Sampling with OPES-MetaD

Enhanced sampling simulations on LDA coordinates were performed using Well-tempered Metadynamics (WT-MetaD) and On the Fly Probability Enhanced Sampling-Metadynamics (OPES-MetaD) as implemented in PLUMED.^32−35^

WT-MetaD works by adding a bias formed from a history dependent sum of progressively shrinking Gaussian hills.^36,37^ The bias at time *t* for CV value *Q*_*i*_ is given by the expression
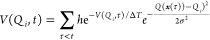
7where *h* is the initial hill height, σ sets the width of the Gaussians, and *ΔT* is an effective sampling temperature for the CVs. Rather than setting *ΔT*, one typically chooses the bias factor γ = (*T* + *ΔT*)/*T*, which sets the smoothness of the sampled distribution.^36,37^ Asymptotically, a free energy surface (FES) can be estimated from the applied bias by (37,38) or using a reweighting scheme.^37,39^

In contrast to the use of sum of Gaussians in traditional MetaD, OPES-MetaD applies a bias that is based on a kernel density estimate of the probability distribution over the whole space, which is iteratively updated.^34,35^ The bias at time *t* for CV value *Q*_*i*_ is given by the expression
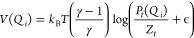
8Here in the prefactor, *T* is the temperature, *k*_B_ is Boltzmann’s constant, and γ is the bias factor. *P*_*t*_(*Q*) is the current estimate of the probability distribution, and *Z*_*t*_ is a normalization factor that comes from integrating over sampled *Q* space. Finally,  is a regularization constant that ensures the maximum bias that can be applied is *ΔE*. For one of our systems, we found that limiting the maximum bias using OPES-MetaD helped prevent unphysical exploration along our LDA coordinate (this is also possible using other approaches such as Metabasin Metadynamics^40^). Even with this limitation, we apply additional wall potentials to prevent exploration well beyond the LDA values for each of our two states. As in WT-MetaD, *F*(*Q*) can be directly estimated from *V*(*Q*) by  or through a reweighting scheme.^35^ Details of the sampling parameters used for each system are given in Sec. 5.

### 2.5 Implementation

Clustering and iterative alignment of trajectory frames prior to learning LDA vectors is performed using our shapeGMMTorch package, which is a high performance version of the methods from ref (25), implemented with pyTorch(41) for accelerated computation on GPUs. shapeGMMTorch is available from https://github.com/mccullaghlab/shapeGMMTorch and can easily be installed in python using the command pip install shapeGMMTorch. We have also created a wrapper library for the training of LDA vectors directly from positional data, which is available from https://github.com/mccullaghlab/pLDA and which can be easily installed with pip install posLDA (although this wrapper was not used in the analysis performed in this paper as it was not yet available). Within posLDA, vectors are learned using the SVD implementation of the scikit-learn LinearDiscriminantAnalysis package.^42^

In order to compute and bias these vectors on the fly within MD simulations, the optimal alignment and linear combination procedure has been implemented in the PLUMED open source library.^32,33^ All procedures, analysis for every case studied in this work, and PLUMED code are made available at https://github.com/hocky-research-group/posLDA_paper_2023, and the code for computing LDA coordinates and Mahalanobis distances on positions will be contributed as a module to PLUMED shortly.

## 3 Results and Discussion

### 3.1 LDA Is a Good Reaction Coordinate for HP35 Folding

In previous work, we applied our shapeGMM clustering approach to a 305 μs trajectory of a 35-amino acid fast-folding folding mutant Villin headpiece domain (HP35), obtained from the D.E. Shaw Research Group.^43^ From our data, we choose to study a six state representation of the data, whose states produce an interpretable representation of folding and unfolding, and which is found not to be overfit by a cross-validation approach. Details of the clustering and cross-validation are provided in ref (25). The definition of this six state model, {**μ**_***i***_, **Σ**_***i***_}_*K*=6_, was trained from 25,000 frames out of ∼1.5 million, and then each frame was assigned to a cluster based on which center was closest in terms of Mahalanobis distance on positions.

**Figure 1:**
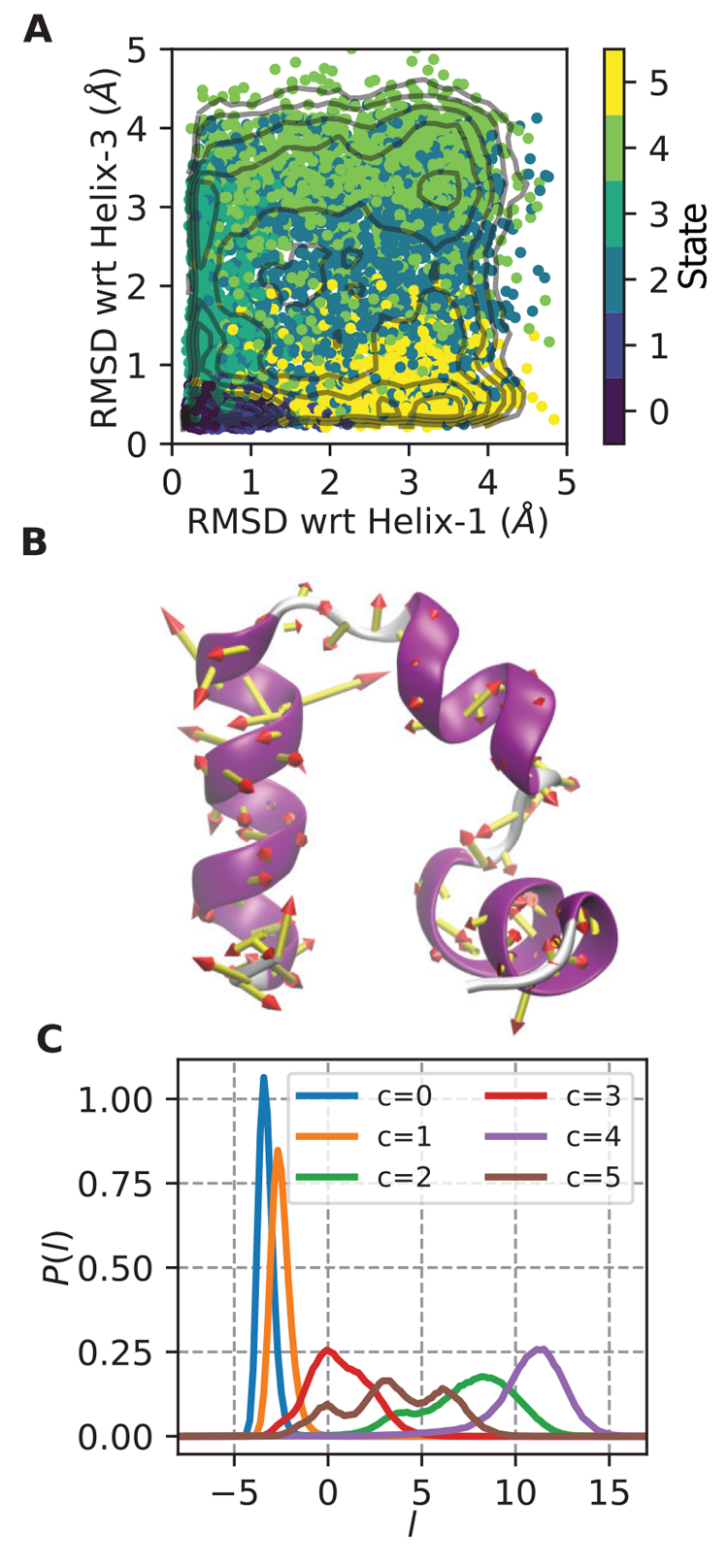
Folding/unfolding coordinate for HP35. (A) Points from HP35 trajectory are colored by state assignment and mapped into natural folding coordinates of the RMSD of residues in helix 1 or helix 3 to that in the folded state (which is a 3 helix bundle). State 0 is the most folded state, and state 4 is the most unfolded state. Contours shown are every 0.5 kcal/mol in the range (0,6). (B) Porcupine plot showing the magnitude of the LDA coefficients trained only on states 0 and 4 from A, overlaid on the starting HP35 structure. (C) Histogram of LDA coordinate *l* for each separate state. *l* evenly separates all states, with states 0 and 4 at maximum separation.

A single folding/unfolding coordinate was constructed by performing LDA on frames assigned to the folded and unfolded states. The folded and unfolded states were assigned based on the RMSD to folded helix 1 and RMSD to folded helix 2 2D map shown in Figure 1A for this long trajectory with points colored by the assigned states. From this figure, we can assign state 0 as the folded state because it is the state with lowest RMSDs (it also has the largest population) and state 4 as the most unfolded state because it is the state with the largest RMSDs. LDA is performed on these two states to produce a single LD vector, denoted *l*, after an iterative alignment of the amalgamated two-state trajectory to the global mean and covariance, as described above. The magnitudes of the coefficients in this vector are illustrated as particle displacement vectors in the porcupine plot in Figure 1B. The histogram in Figure 1C shows the *l* values adopted in each state. We see from these data that this coordinate separates state 0 (*l* ≈ – 3) and state 4 (*l* ≈ 12). To our surprise, this single coordinate, which was trained only on data from state 0 and state 4, separates the other four states as well, which suggests that it might be sufficient to produce transitions between folded and unfolded through physically meaningful configurations.

**Figure 2:**
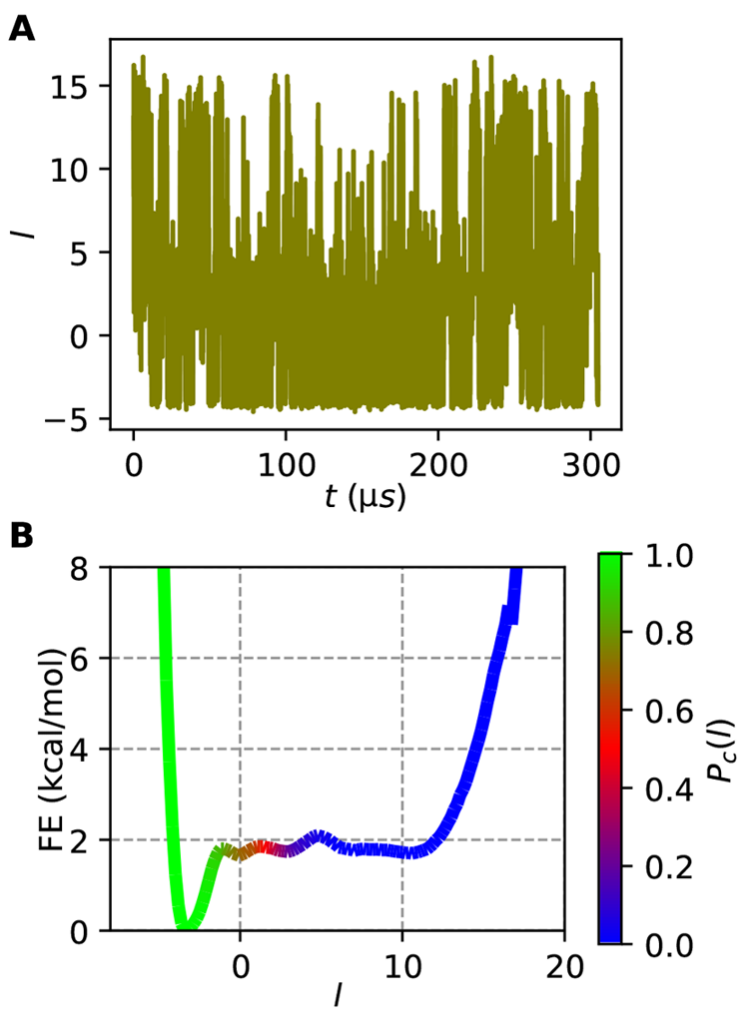
LDA results for the folding/unfolding of HP35 from unbiased MD. (A) LDA coordinate trained on states 0 and 4 vs time for the full 305 *μs* HP35 trajectory shows many transitions between folded (∼−3) and unfolded (∼12) states. (B) Free energy vs *l* for this data, colored by the committor probability in each bin, using 150 bins for the range −8 to 20. This result does not change when discretizing into 50 or more bins.

Figure 2A shows the variation of *l* versus time for this long trajectory and exhibits many transitions between the folded (*l* ≈ −3) and unfolded (*l* ≈ 12) states (for comparison, ref (44) found that this long trajectory contains 61 folding transitions with their definition of folding). In order to assess the quality of this CV, we compute the committor of each frame in the trajectory *c*(***x***_***t***_),^2,45,46^ which for time *t* is 1 if the system reaches a folded state before reaching an unfolded state in the times following *t*.

To assess the quality of a reaction coordinate, we can compute the committor probability for each value of *l* on a grid of size *δl*.
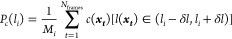
9
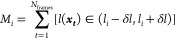
10

In Figure 2B, we show the approximate FES along *l* computed as *F*(*l*) = −*k*_B_*T* ln *P*(*l*) for the long unbiased trajectory, colored by the value of *P*_*c*_(*l*). The FES shows a stable well at a value of *l* = −3 corresponding to the highest population state, the folded one, and very shallow minima for each of the other states. The value of *P*_*c*_ varies continuously from 1 to 0 along this coordinate, reaching a value of 0.5 at *l* = 1, just outside the folded basin. By this metric, our very simple coordinate is a good CV for characterizing the transition between folded and unfolded states, although the lack of a high barrier separating the two states (due to the system being near its melting temperature) makes it more ambiguous how close the point of *P*_*c*_ = 0.5 is to a classic transition state. The coincidence of *P*_*c*_ = 0.5 with a clear barrier is observed in Figure S1 where we train using all 6 states, but for this paper, we chose to focus only on one-dimensional LDA spaces. In Figure S2, we show the FES projected between the folded states and all other states, with each possible choice of alignment.

### 3.2 LDA Is a Reasonable Sampling Coordinate for HP35 Folding

**Figure 3:**
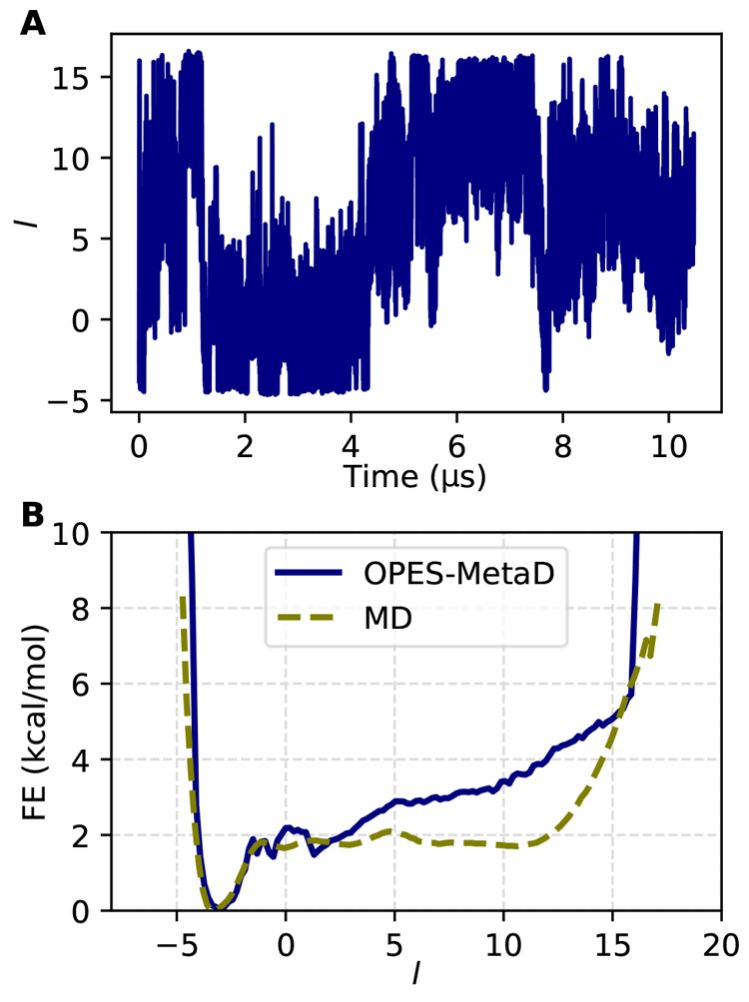
OPES-MetaD sampling on HP35 using the folding/unfolding LDA coordinate. (A) LDA coordinate vs times for OPES-MetaD simulation. (B) Comparison of free energy estimated from unbiased MD and OPES-MetaD.

To assess the ability to sample along an LDA coordinate, we perform OPES-MetaD to bias the system to explore *l* (Figure 3). For the MetaD parameters listed in Sec. 5, we see in Figure 3A that transitions between the folded and unfolded state are accelerated. This corresponds to an estimated FES that is in fair agreement with that obtained from the long unbiased trajectory considering it is obtained in only 3% of the MD time (Figure 3B). Undersampling of the large unfolded region (*l* > 5) is a reflection of the usual problem of sampling slow orthogonal degrees of freedom. Despite this, when we look at the FES projected on natural folding coordinates in Figure S3, we see that our sampling does a good job capturing the main features of the long unbiased trajectory, including the presence of intermediates along the x- and y-axes, and the high energy unfolded state located in the upper right. As inferred from the 1d FES, the most unfolded regions are unexplored, and the statistical weight of the central intermediate basin is incorrect. Shorter replicates of simulations starting from different initial structures (Figure S4) show the variance in FES estimates that could arise if one is not careful to converge sampling. On the whole, our results are evidence that our simple LDA coordinate is a promising first step for sampling between two states of a complex biomolecule.

### 3.3 Accurate Sampling Using LDA for a Bistable Helix

The LDA procedure can be applied to determine a reaction coordinate separating two states even without sampling the actual transition (analogous to ref (6)). To assess this behavior, we investigate the right- to left-handed helix transition of (Aib)_9_, a nine residue peptide formed from the achiral α-aminoisobutyryl amino acid.^47^ The helical states of achiral molecules must by symmetry have equal free energy, and we previously took advantage of this property in benchmarking sampling and clustering methods.^25,48^ The properties of (Aib)_9_ have been characterized in simulation including recently as a tool to benchmark advanced methods for RC optimization.^24,49,50^

**Figure 4:**
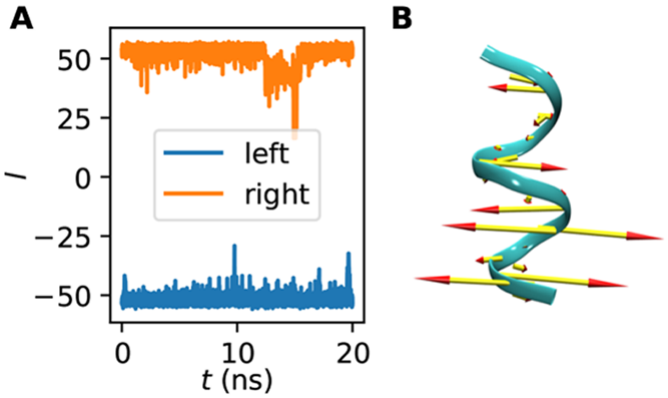
LDA coordinate for helical inversion of (Aib)_9_. (A) LDA coordinate *l* vs time for training data starting from left- and right-handed helixes. (B) Porcupine plot showing the magnitude of the LDA coefficients on the left-handed helical structure.

We performed 20 ns simulations starting from the left- and right-handed states of (Aib)_9_ using inputs from ref (24) (see Sec. 5 for details). We did a three state clustering of the combined MD data (total 40 ns, sampled every ps) and verified that the two most populated clusters are the left- and right-handed states. The coordinates of backbone atoms only were used for the clustering procedure. We then performed an iterative alignment of the combined data to compute a global (**μ**, **Σ**) and then computed a single LDA vector between those frames coming from the left- and right-handed states, respectively from the globally aligned trajectory. Figure 4A shows that this coordinate separates the training data with *l* ∼ 50 indicating a right-handed helix and *l* ∼ −50 indicating a left-handed helix. The left-handed helix is the starting point for further runs (shown in Figure 4B, along with LDA coefficient magnitudes).

**Figure 5:**
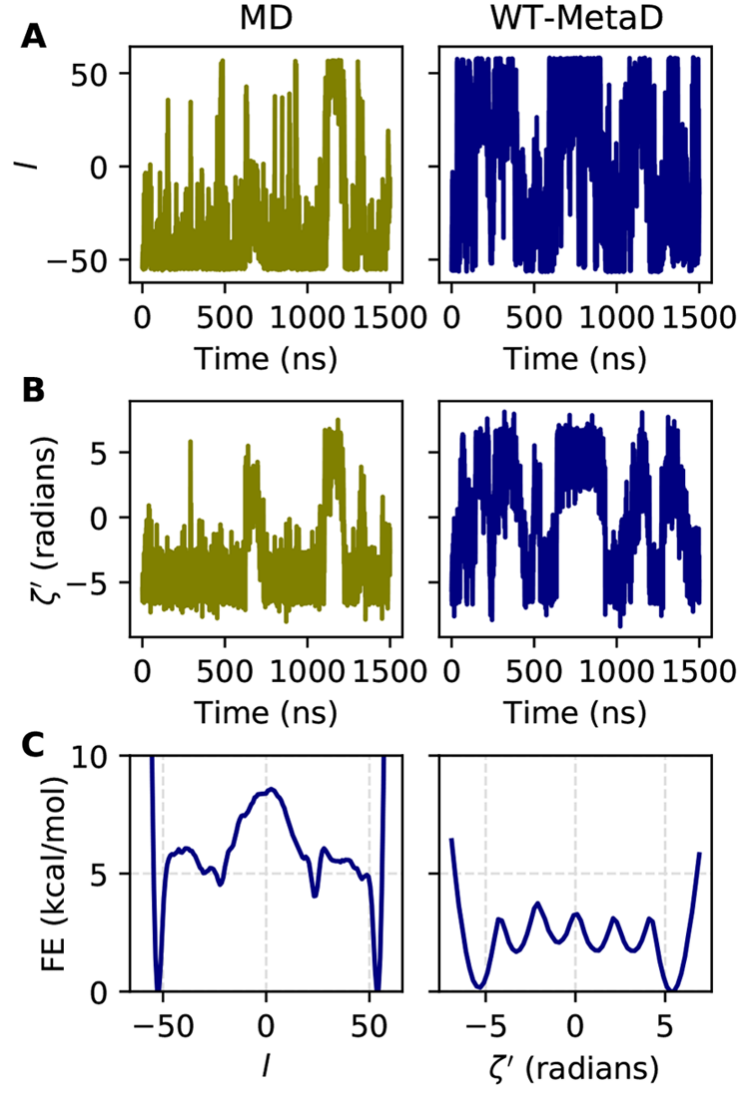
Metadynamics sampling results along the LDA coordinate for (Aib)_9_. (A) LDA coordinate *l* vs time for 1.5 μs of conventional MD and WT-MetaD. (B) Helical parameter ζ vs time for the trajectories in A. (C) FES along *l* and ζ from WT-MetaD simulations.

Having trained *l*, we next performed conventional and WT-MetaD simulations starting from the structure in Figure 4B. Figure 5A shows that MetaD (right) substantially increases the rate of transition between the left- and right-handed states as compared to conventional MD (left).

A more chemically motivated way of computing the helicity of (Aib)_9_ is the parameter ζ′ = −∑_*n*=3_^7^ϕ_*n*_, the negative sum over the central backbone ϕ dihedral angles.^24^ This quantity takes on values of approximately 5 for right-handed and −5 for left-handed helices.^24^Figure 5B shows qualitatively similar behavior for ζ′ as *l*.

Figure 5C shows the FES computed for these two quantities. The sampled *l* has a nearly perfectly symmetrical FES, and in particular the free energy difference between the left- and right-handed states is negligible. For the FES of the nonbiased ζ′ computed by reweighting, the result is nearly as symmetrical, and the offset in free energy between the left- and right-handed size is visible but minuscule. This result appears to be as good as that obtained in ref (24), which uses a very sophisticated iterative process and 900 ns of unbiased and biased simulation data to obtain an optimized sampling coordinate as compared to our 40 ns of input data; however, their optimized coordinate appears to perform better in terms of transitions per unit time generated with their choice of MetaD parameters. As detailed in Sec. 5, the parameters used in our WT-MetaD simulation are very gentle; their magnitude was limited by “crashing”, which typically occurs due to inaccurate numerical integration. To check this, we demonstrate in Figure S5 that use of a 1 fs integration time step allows us to use much more aggressive MetaD parameters, which results in much more frequent transitions, as well as accelerated convergence enough to justify the use of a smaller time step (Figure S6). It is possible that implementation of analytical derivatives for our procedure may further mitigate this issue if they can be properly derived, and we will pursue this going forward.

## 4 Conclusions and Outlook

In this work, we demonstrated that LDA on positions computed from two states of a system produces a good reaction coordinate, both in terms of state transition kinetics and our ability to bias that coordinate to assess the FES along that coordinate. This was true for (Aib)_9_ even though the RC was trained only using short simulations starting in each state, making this a promising approach even when only structures of end points of a process are available. In contrast to ref (6) where input features were internal coordinates, we were able to use standard LDA rather than HLDA in this case and achieve good performance.

We note that LDA on positions would not apply directly to problems such as molecular dissociation since the dissociated states cannot be aligned to a single average structure; however, we do think this coordinate would work well for apo-holo transitions of a biomolecule and could easily be combined with a ligand-distance coordinate to overcome sampling chalenges e.g. as observed in ref (51). There are, of course, difficulties in resolving structural states of globular proteins that could make application of shapeGMM and subsequent LDA challenging. Namely, structural states of globular proteins can differ in only a small fraction of the total degrees of freedom. We feel that the heterogeneous nature of allowed covariance in the Kronecker form of shapeGMM will allow us to resolve these states with adequate sampling. Once the clusters are resolved, the LDA procedure described in the current manuscript will highlight the coordinates relevant to separate the clusters.

For HP35, multidimensional LDA by construction better separates all of the states of the molecule and may also provide an even better reaction coordinate for kinetics (Figure S1). It is not yet clear if this result is general or specific to the HP35 system. Regardless, the use of multidimensional LDA as an RC is intriguing, and we are currently investigating the advantages and limitations of these coordinates. However, this is not an option when information about multiple states is unavailable a priori (such as in the case of (Aib)_9_) which is why we did not include it here. For cases like that, it would be intriguing to first sample along the 1-dimensional reaction coordinate, then train a GMM with a higher number of states, and continue iterating this approach.

The use of states defined from our GMM clustering approach presents both an advantage and disadvantage as illustrated in the case of HP35. Our approach allowed us to explore the folding/unfolding process and most of the conformational landscape (Figure S3), but we were not able to fully sample the FES around the unfolded state. For sampling a broad and entropy dominated state, combining CV based sampling on position LDA coordinates with tempering or temperature accelerated methods should provide more accurate information in this region as in many past studies.^52−56^

In both the case of HP35 and (Aib)_9_, we were able to accelerate transitions between two states using MetaD or OPES-MetaD. In our hands, the biased simulations were sensitive to sampling protocol in terms of being able to run microseconds or longer without “crashing”. HP35 was less sensitive to this issue using OPES-MetaD, while (Aib)_9_ performed better with standard WT-MetaD. For this reason, we initially used small bias factors and hill heights/barrier heights, which resulted in fewer transitions and presumably worse convergence in fixed simulation time. We speculated that some of this sensitivity may come from our choice of the global trajectory mean and covariance as the reference state when computing our LDA vectors; however, subsequent tests using alignment to left- or right-handed helices for (Aib)_9_ showed that these alignments were more sensitive to crashing and had worse convergence performance, supporting our initial choice of global alignment (Figures S7,S8). A compelling option is presented in the ATLAS method of ref (28), where bias is computed along vectors to multiple reference states, weighted by distance from that reference state, and we are beginning to assess that approach.

## 5 Simulation Details

All simulations were performed using GROMACS 2019.6^57^ with PLUMED 2.9.0-dev.^32,33^ GROMACS “mdp” parameter files and PLUMED input files are available in our paper’s github repository for complete details.

### 5.1 HP35 Simulations

A 305 μs all-atom simulation of Nle/Nle HP35 at *T* = 360 K from Piana et al.^43^ was analyzed. The simulation was performed using the Amber ff99SB*-ILDN force field and TIP3P water model. In that simulation, protein configurations were saved every 200 ps, for a total of ∼1.5 M frames. For our simulations, we solvate and equilibrate a fresh system using the same force field at 40 mM NaCl. Minimization and equilibration are performed using a standard protocol (http://www.mdtutorials.com/gmx/lysozyme/index.html), at which point NPT simulations are initiated at *T* = 360 K. mdp files for all steps of this procedure and the topology files are all available from the paper’s github page (https://github.com/hocky-research-group/posLDA_paper_2023).

OPES-MetaD simulations are performed with γ = 8, *ΔE* = 10 kcal/mol, pace of 500 steps, and a multiple time step^58^ stride of 2. Quadratic walls are applied at *l* = 5 and *l* = −15 with a bias coefficient of 125 kcal/mol/Å^2^.

### 5.2 (Aib)_9_ Simulations

Equilibrated inputs for (Aib)_9_ were provided by the authors of ref (24). In brief, simulations used the CHARMM36m force field and TIP3P water.^59^ MD simulations are performed in NPT with a 2 fs time step at *T* = 400 K.

WT-MetaD simulations are performed with *h* = 0.005 kcal/mol, σ = 0.43, γ = 2, and a multiple time step^58^ stride of 2. Quadratic walls are applied at *l* = 70 and *l* = −60 with a bias coefficient of 125 kcal/mol/Å^2^. σ was chosen as the σ_*l*_/3 where σ_*l*_ was the standard deviation in *l* over the 20 ns simulation starting from the left helical state used in the training of the CV.

## Supporting Information Available

Comparison of FE profiles along two state and six state LD1 coordinates; Comparison of different state pairs and alignment choices; Comparison of FES in natural coordinates computed from unbiased and biased MD; Comparison of independent runs with less sampling; Comparison of 1 and 2 fs time steps for (Aib)_9_ simulations; Comparison of sampling efficiency for different alignments in (Aib)_9_ (PDF)
