# Supplementary material for: Reaction Coordinates for Conformational Transitions Using Linear Discriminant Analysis on Positions
Source: J Chem Theory Comput. 2023 May 2;19(14):4427–35. doi: 10.1021/acs.jctc.3c00051 (PMC10373481; doi:10.1021/acs.jctc.3c00051)
Supplement: Supplementary file 1 — ct3c00051_si_001.pdf [file ct3c00051_si_001.pdf]

Supporting information for: Reaction coordinates for conformational transitions using Linear Discriminant Analysis on Positions

Subarna Sasmal,[†] Martin McCullagh,[*,‡] and Glen M. Hocky[*,†]

†*Department of Chemistry and Simons Center for Computational Physical Chemistry, New York University*
‡*Department of Chemistry, Oklahoma State University*

E-mail: martin.mccullagh@okstate.edu; hockyg@nyu.edu

S1 Two state vs. six state LDA

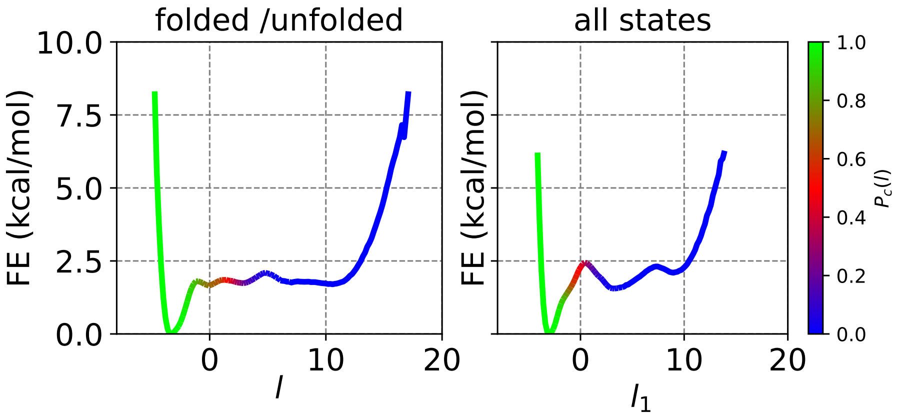

Figure S1: FES obtained from unbiased MD colored by committor probability. The left shows the FES computed along l, the LDA coordinate from states 0 and 4 in our GMM model, and the right shows the FES computed along l_1, the first LDA coordinate from a model trained on all 6 states.

S2 Comparison of different state pairs and alignment choices

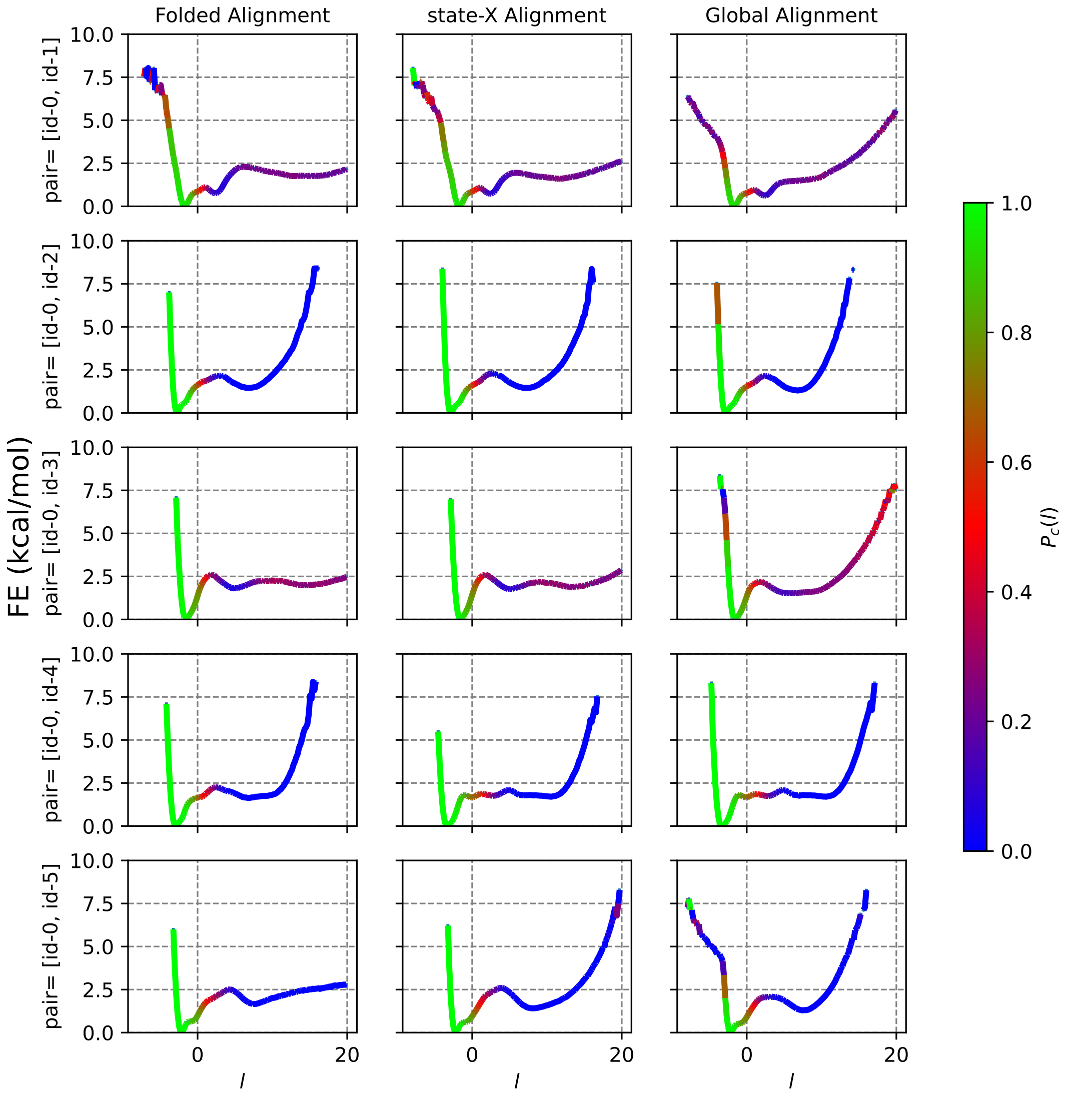

Figure S2: FE profiles along LD1 obtained for three different alignments, considering all possible cluster pairs with the folded cluster (id-0). Each row represents total three FE profiles for a particular cluster pair using: (1) alignment to folded cluster, (3) alignment to cluster X, and (3) global alignment for that cluster pair respectively from left to right.

S3 Comparison of FES in natural coordinates computed from unbiased and biased MD

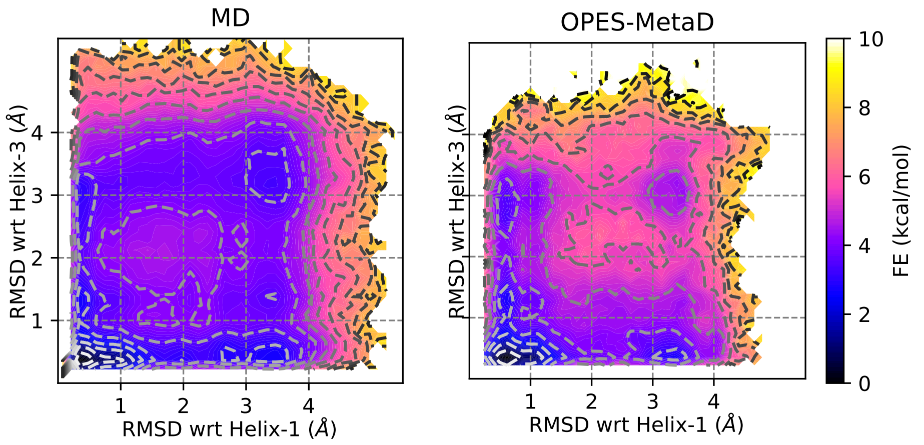

Figure S3: FES from unbiased MD simulation and OPES-MetaD reweighted along coordinates measuring RMSD of the terminal two helices to a reference folded structure.

S4 Comparison of independent runs with less sampling

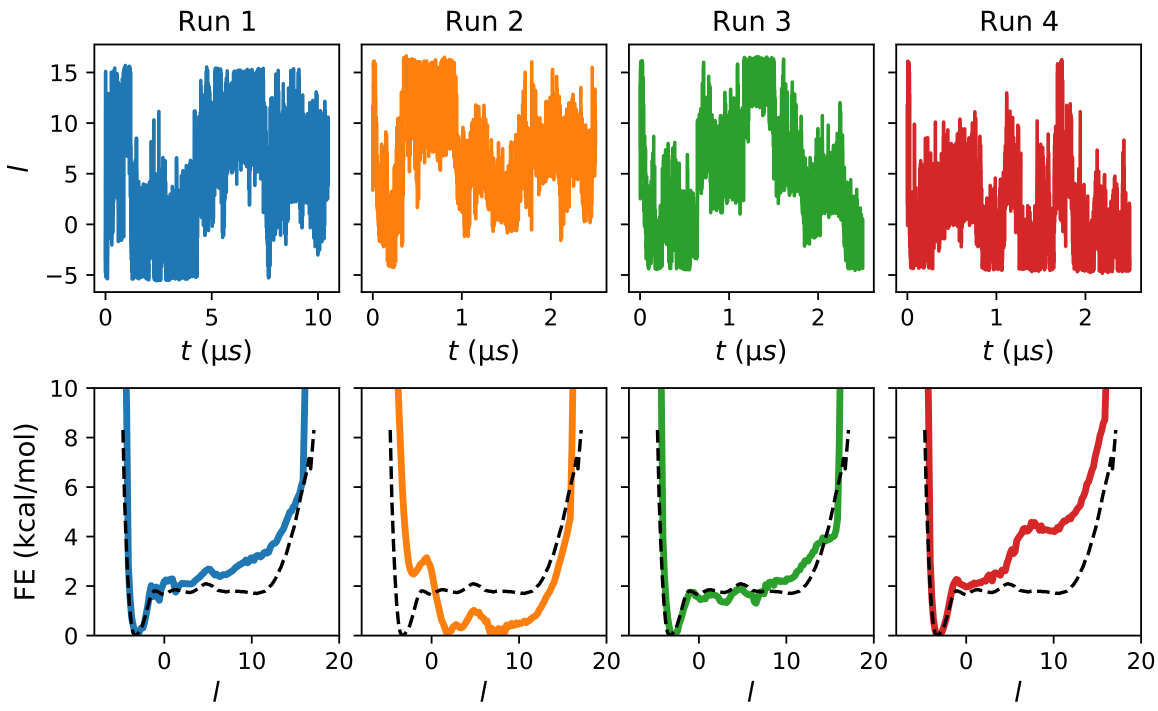

Figure S4: (top) OPES-MetaD replicate trajectories for HP35. Run 1 is the 10μs simulation studied in the main text. The other three runs are 2.5μs runs starting from other points obtained in the original trajectory, with separate equilibrium performed. Each of these three simulations has one to two transitions. (bottom) Comparison of FES obtained from OPES-MetaD for HP35, with a dashed line showing the FES obtained from unbiased MD. Run 3 producing a perfect FES by chance.

S5 Comparing 1fs to 2fs for $(Aib)_9$

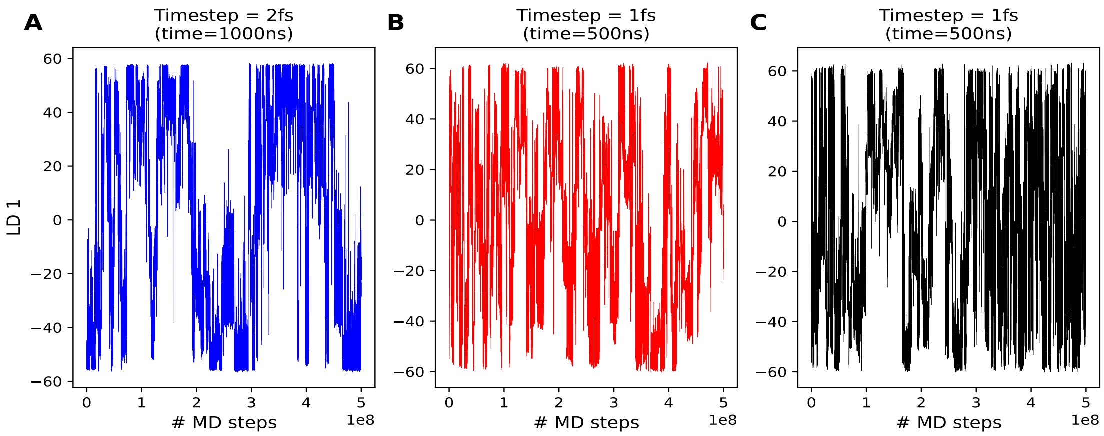

Figure S5: Fluctuation of LD1 coordinate with time from three different simulations (all globally aligned). **A** was performed with h = 0.005 kcal/mol, σ = 0.43, γ = 2 and a multiple time step stride of 2. **B** was performed with h = 0.50 kcal/mol, σ = 0.50, γ = 8 and a multiple time step stride of 2. **C** was performed with h = 1.0 kcal/mol, σ = 1.20, γ = 8 and a multiple time step stride of 2.

S6 Comparing sampling efficiency of different alignments in $(Aib)_9$

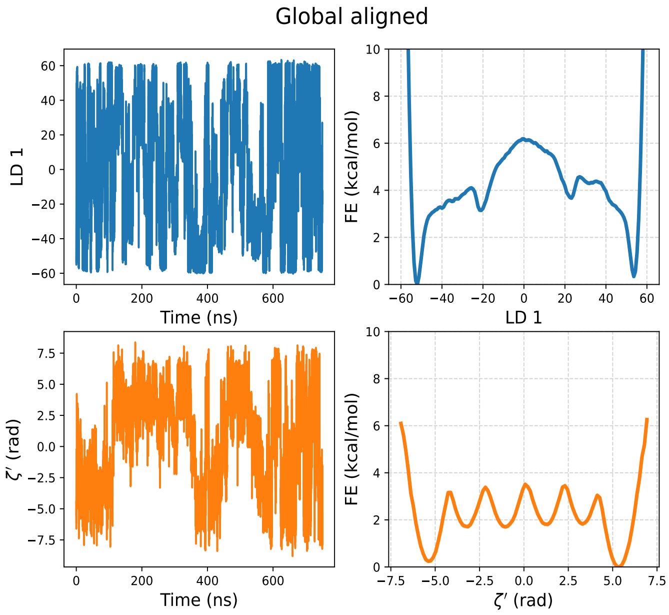

Figure S6: Results from a WT-MetaD simulation using 1fs time step, initiated from left handed state and the global alignment was used. Simulation was performed with h = 0.50 kcal/mol, σ = 0.50, γ = 8 and a multiple time step stride of 2. Top row shows fluctuation of LD1 with time and FE along it. Bottom row shows the same for ζ' coordinate.

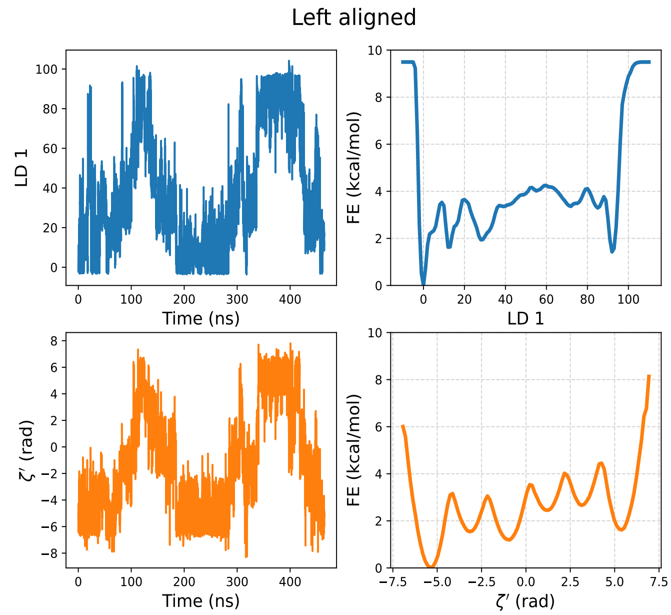

Figure S7: Results from a WT-MetaD simulation using 1fs time step, both initiated and aligned to the left handed helix. Simulation was performed with h = 0.005 kcal/mol, σ = 0.43, γ = 2 and a multiple time step stride of 2. Top row shows fluctuation of LD1 with time and FE along it. Bottom row shows the same for ζ' coordinate. Note that the initial value of LD1 is close to zero according to our definition of LDA coordinate.

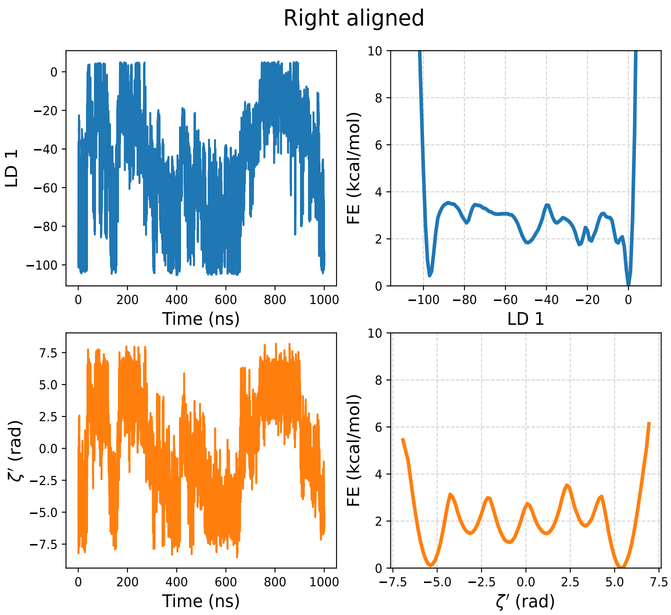

Figure S8: Results from a WT-MetaD simulation using 1fs time step, initiated from left handed state and the system was aligned to the right handed helix. Simulation was performed with h = 0.10 kcal/mol, σ = 1.0, γ = 4 and a multiple time step stride of 2. Top row shows fluctuation of LD1 with time and FE along it. Bottom row shows the same for ζ' coordinate. Note that the initial value of LD1 is far away from zero unlike the case of left alignment.
